# Using behavioral rhythms and multi-task learning to predict fine-grained symptoms of schizophrenia

**DOI:** 10.1038/s41598-020-71689-1

**Published:** 2020-09-15

**Authors:** Vincent W.-S. Tseng, Akane Sano, Dror Ben-Zeev, Rachel Brian, Andrew T. Campbell, Marta Hauser, John M. Kane, Emily A. Scherer, Rui Wang, Weichen Wang, Hongyi Wen, Tanzeem Choudhury

**Affiliations:** 1 Information Science, Cornell University, Ithaca, 14850 USA; 2 Department of Electrical and Computer Engineering, Rice University, Houston, 77005 USA; 3 Psychiatry and Behavioral Sciences, University of Washington, Seattle, 98195 USA; 4 Computer Science, Dartmouth College, Hanover, 03755 USA; 5 Vanguard Research Group, New York, USA; 6 Department of Psychiatry, The Donald and Barbara School of Medicine at Hofstra/Northwell, Hempstead, 11549 USA; 7 Biomedical Data Science Department, Dartmouth Geisel School of Medicine, Hanover, 03755 USA; 8 Facebook, Inc., Menlo Park, USA

**Keywords:** Health care, Medical research

## Abstract

Schizophrenia is a severe and complex psychiatric disorder with heterogeneous and dynamic multi-dimensional symptoms. Behavioral rhythms, such as sleep rhythm, are usually disrupted in people with schizophrenia. As such, behavioral rhythm sensing with smartphones and machine learning can help better understand and predict their symptoms. Our goal is to predict fine-grained symptom changes with interpretable models. We computed rhythm-based features from 61 participants with 6,132 days of data and used multi-task learning to predict their ecological momentary assessment scores for 10 different symptom items. By taking into account both the similarities and differences between different participants and symptoms, our multi-task learning models perform statistically significantly better than the models trained with single-task learning for predicting patients’ individual symptom trajectories, such as feeling depressed, social, and calm and hearing voices. We also found different subtypes for each of the symptoms by applying unsupervised clustering to the feature weights in the models. Taken together, compared to the features used in the previous studies, our rhythm features not only improved models’ prediction accuracy but also provided better interpretability for how patients’ behavioral rhythms and the rhythms of their environments influence their symptom conditions. This will enable both the patients and clinicians to monitor how these factors affect a patient’s condition and how to mitigate the influence of these factors. As such, we envision that our solution allows early detection and early intervention before a patient’s condition starts deteriorating without requiring extra effort from patients and clinicians.

## Introduction

Schizophrenia is a severe and chronic psychiatric disorder with multi-dimensional complex symptoms of hallucinations, delusions, disorganized thoughts, agitated movement, avolition-apathy, and expressive deficit^1^. The symptoms can change both in short periods (e.g. within a day) and long periods of time (over weeks and months) and fluctuate between remission and relapse/exacerbation. It is also considered as a heterogeneous disorder with high variations of symptoms among patients. Pharmacological and non-pharmacological treatments are commonly used to manage symptoms and prevent relapses. To assist clinicians with making clinical decisions for treatments, it is of great importance to develop tools that monitor fluctuations in symptoms and detect early signs of evolving events^2–4^.

Mobile devices have been used to capture users’ behavioral and physiological data to predict users’ mental health conditions^5–7^. For example, in the StudentLife study^8^, Wang et al. collected students’ behavioral data, including sleep, activity, conversation, and location, etc, using smartphones and found strong correlations between the data and the students’ self-reported scores for mental health. Another study showed that location and phone usage data can predict users’ self-reported depression severity scores^9^. Mobility and physical activity data collected using smartphones were also shown to be able to detect clinical depression diagnoses^10^. More importantly, previous work explored the feasibility of using mobile sensing to detect or identify signs and symptoms of schizophrenia. Ben et al.^2^ first explored the feasibility and acceptability of behavioral sensing in outpatients and inpatients with schizophrenia. The CrossCheck study used passive mobile sensing data, including physical activity, sociability, mobility, phone usage, sleep, and characteristics of ambient environments to predict the aggregated self-reported ecological momentary assessment (EMA) scores for 10 different symptom items^11^ and the total scores of monthly 7-item Brief Psychiatric Rating Scale (BPRS) administered by clinicians^12^.

However, the types of symptoms each patient experiences might be different. Clinicians usually need to monitor the change in multiple symptoms in order to assess a patients’ condition. For example, according to *DSM-5*^13^, the criterion for diagnosing the onset of psychotic episodes is whether a patient has experienced two or more key symptoms of psychotic disorder, which include delusions, hallucinations, disorganized speech, grossly disorganized or catatonic behavior, and negative symptoms. As for detecting relapse or exacerbation, even though there haven’t been consistent criteria, clinicians tend to assess if there is any worsening of conceptual disorganization, hallucinatory behavior, suspiciousness, or unusual thought content, etc^14^. Hence, the information on the severity of each symptom is essential for clinicians to assess a patient’s condition, and predicting scores for the individual symptoms will be more informative than just predicting the aggregated scores. Besides, even for the same symptom, the symptom might manifest itself in patients’ behaviors in different ways, which the existing modeling methods have yet to account for. As such, those previously proposed methods still have some limitations in terms of providing clinicians with information for making decisions on delivering intervention and managing patients’ symptoms.

In order to make prediction models provide deeper insights into the change in patients’ conditions, we investigated the relationship between the rhythms in patients’ behaviors and their symptom conditions. Human rhythms are cyclic patterns that recur at regular intervals within humans’ biological systems and behaviors^15^. These rhythms have been developed and evolved to help humans respond to environmental influences, and have been found to have a strong link with mental disorders. Based on the recurring time intervals, these rhythms can be categorized as ultradian, circadian, or infradian rhythm (less than, equal to, or greater than 24 h), and the three different types of rhythms influence people’s mental disorder differently. For example, previous studies suggested that disruption in circadian rhythm leads to numerous psychiatric disorders, such as depression, mania, and schizophrenia^16,17^. As such, some mental health intervention tools have been designed to help people with schizophrenia improve their condition by regulating sleep cycles^18,19^. However, most of these detection and intervention tools predominantly focused on a patient’s circadian rhythm. The information from other rhythms has yet to be fully utilized.

Apart from choosing a new set of features that provide better interpretability, we also trained models that predict scores for the individual symptoms instead of predicting the aggregated scores. This allows better tracking the aforementioned different symptoms. To capture the markers associated with changes in patients’ symptoms and to account for individual differences in the meantime, we trained our prediction models using multi-task learning (MTL). MTL is a method aimed to train machine learning models that provide inferences for multiple related tasks simultaneously while accounting for the similarities and the differences across the tasks^20–28^. In other words, MTL leverages information from different tasks to find a common subset of most predictive features, while the learned weights for those features may be different among models for the different tasks in order to account for inter-task differences. Recent work has applied MTL to multi-modal sensing data to predict users’ level of stress and depression^29^. Taylor et al.^7^ leveraged MTL to account for inter-individual differences in the relationship between behavior and physiology measured with surveys, wearable sensors and mobile phones, and resulting mood and well-being. Specifically, the authors predicted the well-being for a subgroup of people who shared similar personality traits and behaviors by treating the prediction for these people as different tasks. A more recent work by Lu et al.^29^ developed a MTL method to jointly model sensing data collected from different smartphone platforms (Android and iOS) for depression detection, where predicting self-reported assessment scores and clinical severity of depression on each platform were considered four different tasks. Taken together, the aim of this paper is to predict more fine-grained symptom trajectories of schizophrenia in terms of patients’ self-reported EMA scores using clinically meaningful rhythm features—the different types of cyclic patterns in patients’ behaviors and their surrounding environment that are extracted from their passive mobile sensor data. As such, patients’ conditions can be automatically assessed at a granular level without relying on them self-reporting.

The contribution of this work lies in investigating how rhythm features and MTL can be used in tandem to make our prediction models provide more fine-grained information on the different dimensions of schizophrenia symptoms by accounting for the heterogeneity in patients’ symptoms. Our results showed that MTL models perform statistically significantly better than the models trained with non-MTL methods in predicting a patient’s individual symptom trajectories, such as feeling depressed, social, calm, and hearing voices. In addition, we showed how these rhythm features can be grouped based on different levels of granularity to allow better interpretability of the relationship between patients’ behavioral patterns and their symptoms. Such information can potentially be beneficial for designing intervention technologies regarding when and how interventions should be delivered to prevent patients’ conditions from deteriorating.

## Results

### Predicting EMA scores

**Figure 1:**
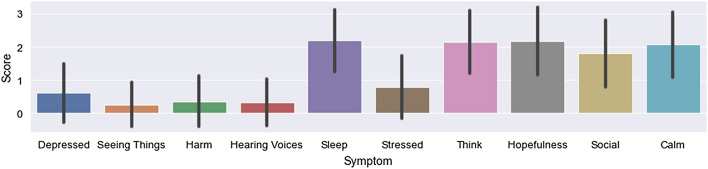
Summary of the individual EMA scores. The error bars represent the standard deviations.

**Figure 2:**
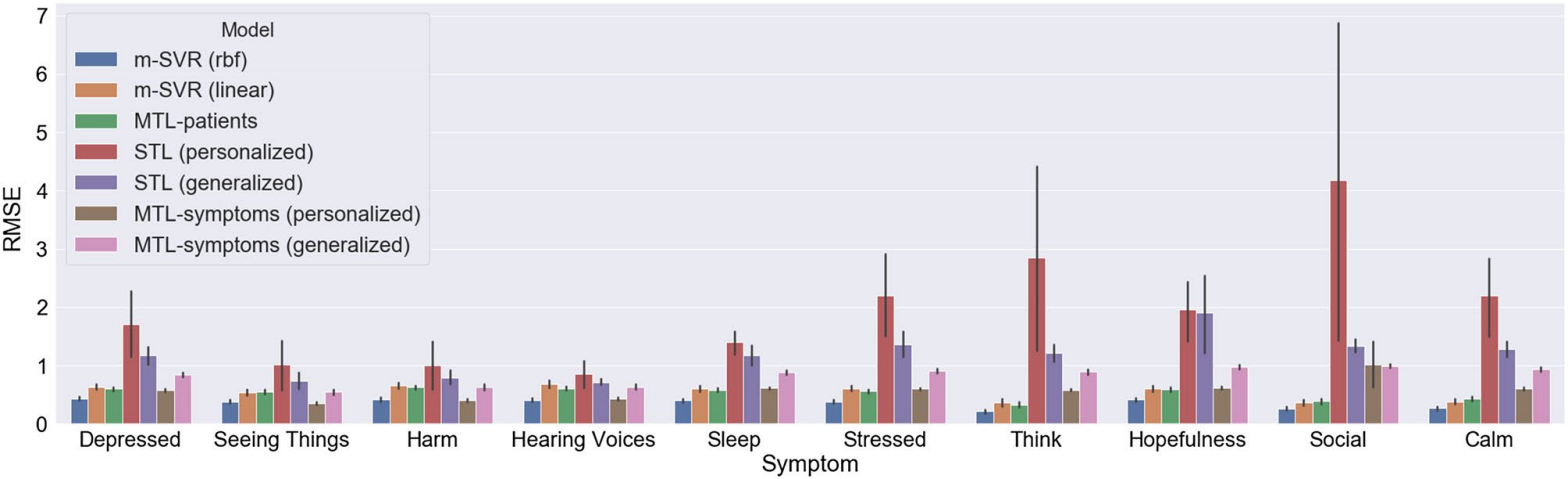
Comparison of the mean root-mean-square errors (RMSE) of different prediction models. The error bars represent the $95\%$ confidence intervals.

A summary of the patients’ self-reported scores for each EMA item, including mean and standard deviation, is shown in Fig. 1. We first compared the root mean square error (RMSE) of all the different machine learning models for each of the schizophrenia symptoms (Fig. 2). Overall, models trained with multi-output support least-squares vector regression machines with RBF kernel (m-SVR-RBF) had the lowest mean RMSE (12$\%$ error rate). It is worth noting that the personalized STL models had a mean RMSE larger than the range of EMA scores, namely 0–3). The reason is that the way linear models give predictions is by computing the inner products between the feature weights and the feature values (and adding intercepts). Since the weights of these models are based on the distribution of the training data, the models are likely to have larger prediction errors if the distributions of the training data and test data (which was held out during the training) are very different. This is very often the case in the data from individuals with mental illnesses, particularly when they are in psychotic episodes.

**Figure 3:**
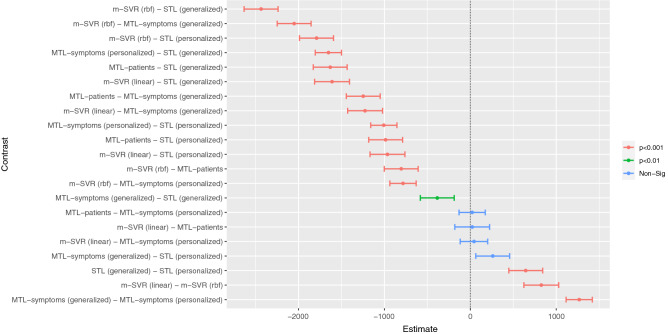
Post-hoc Tukey HSD pairwise comparisons of the mean RMSEs of the individual algorithms. The error bars represent the $95\%$ confidence intervals.

The result of the aligned rank transform ANOVA with algorithm and symptom as the main effects suggests that both algorithm ($\textit{F}(6, 6360) = 231.3940, \textit{p} < 0.001$) and symptom ($\textit{F}(9, 6360) = 60.2962, \textit{p} < 0.001$) have statistically significant effects on the prediction error. Additionally, the results of the post-hoc pairwise comparisons of all the different algorithms (Fig. 3) show that all the MTL algorithms performed significantly better than all the STL algorithms, except there was no statistically significant difference between generalized MTL models and generalized STL models (Generalized models are models that are trained on data from multiple users. Please refer to section Algorithms for the more details about generalized MTL and STL models.). This confirmed our hypothesis that the information from either other patients or other symptoms can improve the prediction accuracy.

Next, when evaluated on the chronologically withheld test data (on average 83.4 samples (S.D. = 37.6) and 20.9 samples (S.D. = 9.39) in the training and testing folds respectively), the m-SVR(rbf) models had a median RMSE of 0.309 ($10.3\%$ of the scale) compared to the median RMSE of 0.314 ($10.5\%$ of the scale) when evaluated on randomly withheld test data, while MTL-patients models showed a median RMSE of 0.535 ($17.8\%$ of the scale) tested on chronologically withheld test data compared to the median RMSE of 0.505 ($16.8\%$ of the scale) when tested on withheld test patients’ data. No statistically significance in the median RMSE was found using the different cross-validation procedures for both m-SVR(rbf) models ($\textit{Z} = 0.32145, \textit{p} = 0.75$) and MTL-patients models ($\textit{Z} = 0.66711, \textit{p} = 0.50$).

**Figure 4:**
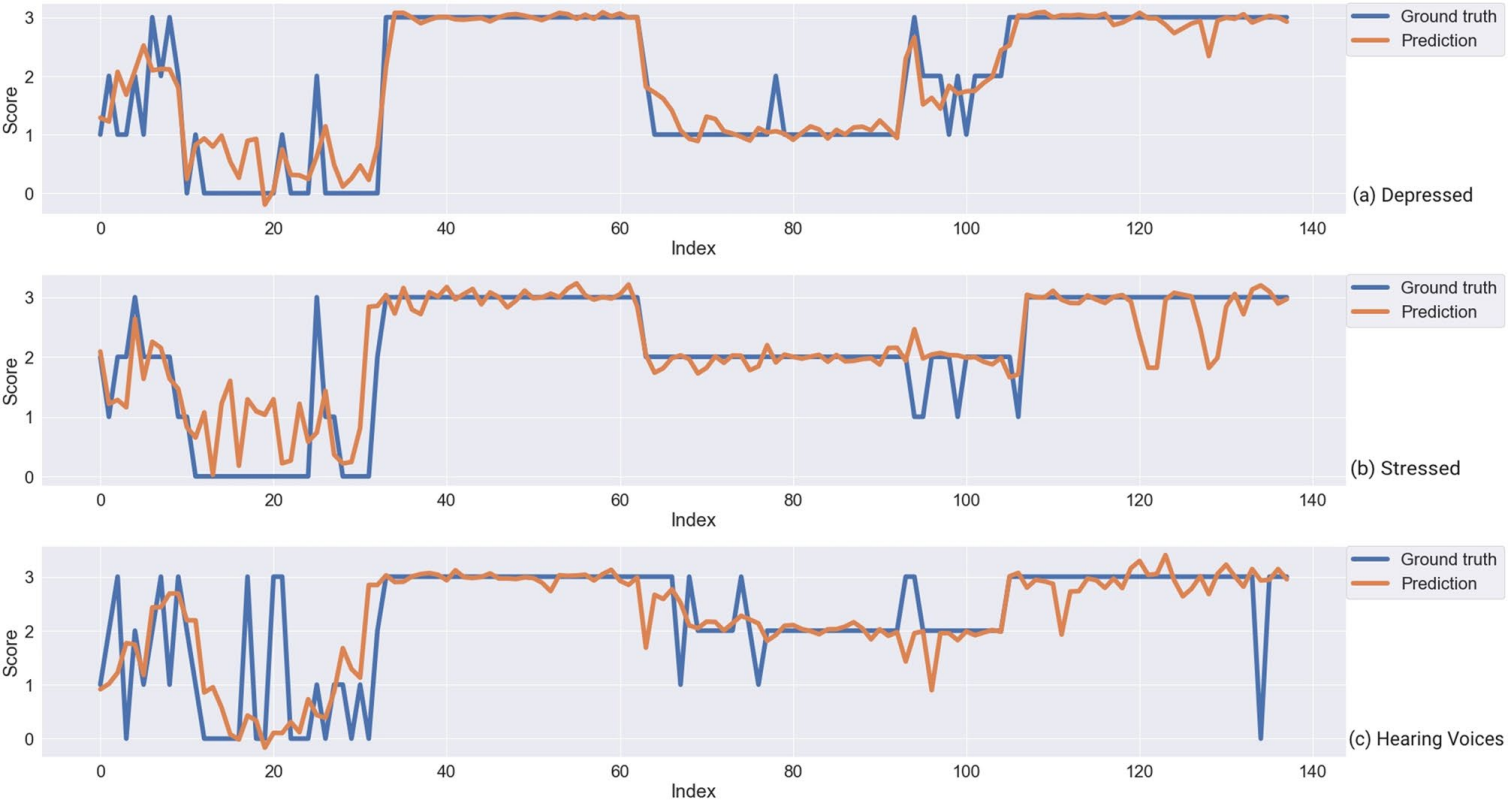
Predicted symptom trajectory of one patient for depressed, stressed, and hearing voices by MTL-patients models and the ground truth (index represents the chronological order of each EMA response).

**Figure 5:**
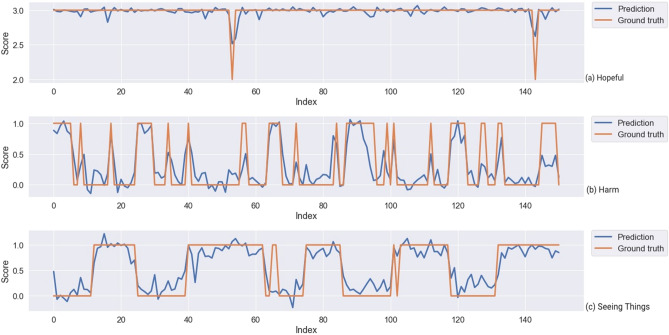
Predicted symptom trajectory of one patient for hopeful, harm, and seeing things by m-SVR (RBF) models and the ground truth (index represents the chronological order of each EMA response).

Finally, the results of the paired tests showed that our rhythm features resulted in statistically significantly lower median RMSE for EMA depressed ($\textit{Z} = -2.6, \textit{p} = 0.037$), hearing voices ($\textit{Z} = -3.372, \textit{p} = 0.0045$), stressed ($\textit{Z} = -3.0221, \textit{p} = 0.00251$), think clearly ($\textit{Z} = -6.8212, \textit{p} < 0.001$), and feeling social ($\textit{Z} = -5.9514, \textit{p} < 0.001$), while the median RMSE statistically significantly increased for EMA harm (*Z* = 3.5073, *p* = 0.0032) and sleep (*Z* = 3.7297, *p* = 0.0015) after using rhythm features. This suggests that rhythm features not only provide better interpretability but even allow more accurate prediction for symptom depressed, hearing voices, stressed, think clearly, and feeling social, which is useful for tracking symptom changes over time. Figures 4 and 5 are examples of predicted trajectories for different symptoms by MTL-patients and m-SVR respectively.

### Heterogeneity

Beyond predicting the symptom scores, we also analyzed the top predictive features in the MTL-patients models for the individual symptoms to see if the models can provide some insights into digital markers that clinicians can utilize as related to different symptoms. The features are ranked based on the features’ weights in the MTL-patients models. The higher the weights are, the more predictive the features are (see the top predictive features in the tables in the Supplementary File).

We found that different symptoms have different sets of top predictive features. For example, multiple-scale entropy of ambient sound with a longer window length has greater influence on symptom Harm and Voice, which means that greater variation in the ambient sound, or noise, over a longer period of time is likely to exacerbate Hearing Voices. And the power spectrum density of text messaging patterns with a shorter window length is more predictive of symptom Think Clearly and Stressed, which suggests that more abrupt change in text messaging pattern during a short period of time is associated with higher levels of stress.

**Figure 6:**
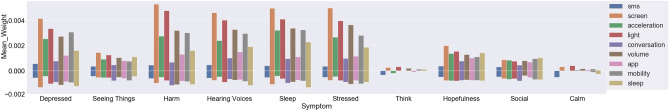
The mean feature weight for each modality for different EMA items (the values for the mean positive and negative weights are plotted separately).

**Figure 7:**
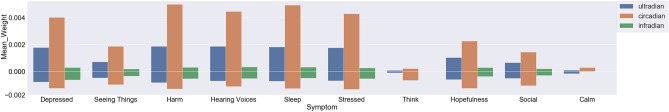
The mean feature weight for each periodicity for different EMA items (the values for the mean positive and negative weights are plotted separately).

**Figure 8:**
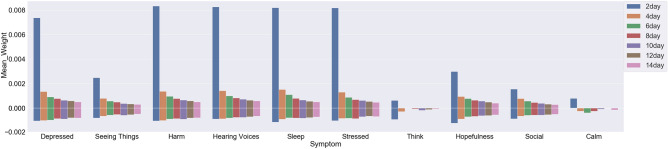
The mean feature weight for each window length for different EMA items (the values for the mean positive and negative weights are plotted separately).

When comparing the importance of the different factors within each of the dimensions after feature grouping, we found that, for dimension *sensor modality*, phone usage (screen lock/unlock) features overall have the highest importance for predicting Feeling Social, Feeling Calm, and Sleep, followed by ambient light (Fig. 6); however, the difference is not significant, which suggests that there is some heterogeneity in how the changes in patients’ symptoms manifest in their behaviors and environments. As for dimension *periodicity*, circadian rhythm related features are more predictive of symptom changes ($p<0.01$ ) than features of the other rhythm types (Fig. 7). In addition, when investigating the mean absolute values of features with positive weights and negative weights, we found that both the positive-weight and negative-weight circadian rhythm features have a similar influence on the score prediction for Feeling Hopeful and Feeling Depressed, which suggests that these symptoms might be more prone to the change in a patient’s circadian rhythm. Finally, for dimension *window length*, features computed with 2-day window, the smallest window, are more predictive of the symptom scores than features computed with the other window lengths ($p<0.01$), which suggests that more recent data might be more predictive of symptom changes (Fig. 8).

### Subtypes

**Figure 9:**
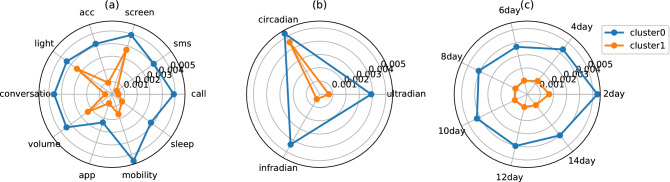
Characteristics of patients of two subtypes for symptom Depressed after applying K-Means clustering to the absolute feature weights and computing the mean absolute weights for each category. The radar charts show the mean aggregated feature weights for different (**a**) modalities, (**b**) periodicities, and (**c**) time-window in each subtype.

Figure 9 is an example of different subtypes for symptom Depressed, identified by clustering weights in MTL-patients models. Two different subtypes were identified (mean silhouette score = 0.56), with 3 patients in one cluster, subtype-0 (mean Depressed score $= 1.1, S.D. = 0.40$) and 56 patients in the other cluster, subtype-1 (mean Depressed score $= 1.8, S.D. = 0.74$). Subtype-0 showed statistically lower scores in Think than Subtype-1 (mean Think score$= 0.02, S.D. = 0.02$ in Subtype-0 and mean score=0.27, *S*.*D*. = 0.45 in Subtype-1).

And the two subtypes have quite distinct top features for predicting Depressed (Table 1). Generally speaking, subtype-0 patients are influenced more by their phone usage pattern, whereas subtype-1 patients are influenced more by the environmental light. When looking at the contribution of different factors in sensor modality, periodicity, and window length respectively, we found that the mean aggregated weight for the individual factors is statistically larger for subtype-0 patients than for subtype-1 patients ($p<0.05$ with Bonferroni correction). The results suggest that subtype-0 patients may be more prone to disturbance in either their behavioral pattern or their environment. The same amount of change will result in more prominent fluctuation in their symptoms. Moreover, for different subtypes, the role of different factors also differs. For subtype-0, different factors have relatively similar contribution, while for subtype-1, some factors (phone usage, ambient light, and ambient noise for instance) play a more influential role than the other factors.

**Table 1:** The top 10 predictive rhythm features and the associated mean feature weights for two different subtypes for symptom Depressed.

Subtype 0	Weight	Subtype 1	Weight
#missed_calls$\bigotimes$16-hour_PSD$\bigotimes$12-day_window	0.046	light$\bigotimes$amplitude$\bigotimes$2-day_window	0.058
#SMS_sent$\bigotimes$32-hour_PSD$\bigotimes$14-day_window	0.042	light$\bigotimes$amplitude$\bigotimes$14-day_window	0.057
conversation_length$\bigotimes$20-hour_PSD$\bigotimes$14-day_window	0.041	light$\bigotimes$amplitude$\bigotimes$12-day_window	0.057
#incoming_calls$\bigotimes$36-hour_PSD$\bigotimes$14-day_window	0.038	light$\bigotimes$amplitude$\bigotimes$8-day_window	0.057
screen_on_time$\bigotimes$32-hour_PSD$\bigotimes$8-day_window	0.036	light$\bigotimes$amplitude$\bigotimes$10-day_window	0.057
screen_on_time$\bigotimes$32-hour_PSD$\bigotimes$14-day_window	0.036	light$\bigotimes$amplitude$\bigotimes$6-day_window	0.056
screen_on_time$\bigotimes$mean_deviation$\bigotimes$2-day_window	0.032	light$\bigotimes$amplitude$\bigotimes$4-day_window	0.056
#SMS_read$\bigotimes$10-hour_PSD$\bigotimes$4-day_window	− 0.032	screen_on_time$\bigotimes$amplitude$\bigotimes$6-day_window	0.051
#outgoing_calls$\bigotimes$16-hour_PSD$\bigotimes$10-day_window	0.031	screen_on_time$\bigotimes$amplitude$\bigotimes$8-day_window	0.051
screen_on_time$\bigotimes$median_deviation$\bigotimes$6-day_window	0.030	screen_on_time$\bigotimes$amplitude$\bigotimes$4-day_window	0.050

The naming of the features follows the format [*Modality*]$\bigotimes$[*Rhythm Metric*]$\bigotimes$[*Window Length*], which denotes the modality of the sensor data, the rhythm metric, and the window length used for extracting the feature.

## Discussion

In this paper, we focus on developing models that can provide more interpretable and granular information on symptoms of schizophrenia. To this end, we applied novel approaches to both our feature engineering and model training. We first applied a variety of rhythm metrics to extract human-interpretable rhythm features. Then, we employed multi-task learning to train prediction models that can account for the heterogeneity in different patients and symptoms when predicting the symptom scores. In this section, we discuss the findings and the implications of our results and how these models with better interpretability can be used for early interventions.

### The link between rhythms and schizophrenia symptoms

Human rhythms, particularly circadian rhythm, have been shown to have strong relationships with schizophrenia. However, the relationships between other types of rhythms, namely ultradian rhythm or infradian rhythm, and schizophrenia have not been well studied yet. To this end, we aim to expand the current understanding of how these different rhythms impact people with schizophrenia at different levels.

First, at a more coarse level, we grouped the rhythm features based on ultradian, circadian, and infradian rhythm, and looked at the importance of the individual rhythms for predicting different schizophrenia symptoms. We found that circadian rhythm has a great influence on symptoms of schizophrenia, particularly Sleep, Feeling Social, and Feeling Calm. This corroborates the findings in the literature regarding the role of circadian rhythm in people’s sleep^30^ and social functioning^31^. Beyond circadian rhythm, ultradian rhythm also has an influence on the symptoms of depression and hallucination, such as Seeing Things and Hearing Voices. People with depression are known to have greater amplitude in the ultradian cycle of their mood than healthy people do^32^. Our results further suggest that ultradian rhythm also manifested in patients’ passive sensing data, and their depressed mood may be susceptible to the change in their ultradian rhythm. And for the symptoms of hallucination, the presence of repetitive^33^ and agitated behaviors^34^ due to hallucination may result in the disturbance in their ultradian rhythm. As such, the change in ultradian rhythm can be an indicator of whether a patient is hallucinating.

Next, at a more granular level, we looked at the influence of the interaction between different types of rhythms and different sensor moralities. For example, we found that the ultradian rhythm of ambient sound has a great influence on symptom Hearing Voices and Feeling Harm. Previous studies have shown that environmental noise has adverse effects on the cognitive performance of people with schizophrenia^35,36^. Our results further suggest that the variations in the ambient noise in a period of 3–5 h may have more pronounced effects on hallucinations. Another example is the interaction between ultradian rhythm and text messaging pattern. Ultradian rhythm of text messaging pattern with period of 8–12 h has the most prominent influence on Think Clearly and Feeling Stressed, which means, just like having breakfast, lunch, and dinner, people also have repeated patterns of text messaging throughout the day. In addition to the relationship between text messaging and increased level of stress^37–39^, the results also suggest that the change in the text messaging pattern will also affect the level of stress and anxiety.

Taken together, these different types of rhythms provide a more intuitive way to interpret the relationships between a patient’s behaviors and their symptoms. At a high level, the different rhythms are good indicators of a patient’s general condition, such as whether they are experiencing hallucinations and whether they have clear thoughts, while the rhythms in certain types of sensor modalities can provide more detailed information on specific symptoms. This can help determine when and the type of intervention to be delivered to avoid certain symptoms or prevent them from worsening. For example, if there is an unusual change in the ultradian rhythm of environment noise for a couple of hours, the system can prompt the patient to move to an environment that has a lower and more stable level of ambient noise to prevent the noise from affecting the patients’ cognitive performance. If the system notices that the patient’s phone usage in certain periods, for example in evening, has a very different pattern than in other periods (morning and afternoon), the system can intervene to change the patient’s phone usage pattern, delaying the arrival of phone notifications for instance, to avoid an increase in stress.

### The role of multi-task learning

From our results, we found that models trained with multi-task learning performed statistically significantly better than the models trained with single-task learning, which confirms our hypothesis that multi-task learning can help achieve better prediction accuracy by accounting for the heterogeneity in different patients and different symptoms. Especially, models trained with single-task learning generally have larger standard errors, which suggests that it is harder for models to capture the entire variability in a patient’s behavioral pattern associated with each symptom due to the limited number of training instances and is more likely to over-fit. In addition, there is no statistically significant difference between the performance of MTL-patients models and the performance of MTL-symptoms models, which suggests that information from either other symptoms or other patients can be both useful for finding latent variables and improving model performance. It is worth noting that the results suggest that non-linear MTL models (i.e., m-SVR(RBF) models) in general have higher prediction accuracy than linear models, such as MTL-patients. In order words, there is a trade-off between prediction accuracy and interpretability. Decisions on which type of MTL models should be used will depend on what clinical applications or settings the models will be deployed for. If the goal is just to track the symptom trajectories as a monitoring tool, then m-SVR models would be a good option. However, if the goal is to provide interventions, then being able to interpret the changes in certain rhythms with respect to the changes in certain symptoms is of great importance. Further, MTL-patients models can be particularly beneficial to model deployment in practice. To train and deploy a model for a new patient, the amount of data from the patient can be reduced by leveraging information from a set of existing patients, which in turn reduces not only the burden on the patient but also the duration of data-collection before the model is ready to be deployed.

### The effect of historical information

Another goal of this work is to investigate the amount of data needed for predicting the trajectories of different symptoms. For some symptoms, the change may be more pronounced in patients’ passive sensor data, whereas for other symptoms, changes may be more gradual and more data is needed in order to detect those changes as a result. In our study, we found that rhythm features computed based on data from the past two days are more predictive of changes in patients’ feelings about being social and calm, whether they slept well, and whether they experienced hallucinations. On the other hand, detecting changes in a patients’ feelings about hopefulness, depression, and harm may require data from a longer period of time. Determining the right amount of historical data needed for predicting the individual symptoms will save clinicians’ time for making clinical decisions by presenting clinicians the most relevant information. More specifically, if a clinician wants to look at whether there was any sign of a patient having hallucinations, information on any behavioral irregularity during the past two to three days can be presented to the clinician. On the other hand, if a clinician wants to look for traces indicative of symptoms of depression, then the historical information during the past one to two weeks should be presented.

### Heterogeneity in different participants

Despite the fact that combining data from multiple participants can help a model better capture the variability in behaviors that causes the changes in their symptom trajectory, we found that there is heterogeneity in different patients. Some participants are more prone, or sensitive, to certain rhythm changes than the others. The same amount of rhythm change may cause these patients’ symptoms to change in different degrees. By knowing which subtype a patient belongs to, the clinician can suggest them take some preventive measures to avoid getting exposed to those stimuli. For example, for patients who are particularly sensitive to the rhythm change in the ambient light and sound, the clinicians can suggest that their patients avoid going to environments that have stimulating lights and sounds. For patients who are more prone to ultradian rhythm changes, the clinician can suggest those patients develop and follow regular daily routines to avoid disturbance in their ultradian rhythm.

### Potential clinical use

Ultimately, the entire pipeline is aimed to provide clinicians with more interpretable and actionable information on a patient’s condition without requiring frequent clinical checkups, nor relying on patients’ self-reports. As a result, it can potentially provide early detection and early intervention.

#### Early detection

In addition to patients’ visits with their clinicians, with our system, clinicians can monitor how the patients are doing in between visits to the clinics. In addition, the patient-to-clinician ratio is generally high, which means that a clinician usually has to take care of multiple patients, with brief and/or infrequent visits. Therefore, clinicians do not have time to go through all of the information provided by the traditional prediction models in order to best evaluate patients’ conditions. Our system can show clinicians the predicted score for each of the symptoms, which will give them an overall idea about patients’ conditions in different dimensions.

If a clinician needs more information than just predicted symptom scores in order to help determine whether they should give a patient further examination, the system can quickly present the summarized information on the patient’s recent behaviors, such as the patterns of their rhythms. Beyond the high-level summarization, the system can also present more granular information by zooming in on the rhythms of particular behavioral patterns based on the specific domains that the clinician wants to examine, such as their physical activity, text messaging patterns, or even the pattern of their environmental stimuli.

#### Early intervention

Providing real-time feedback and intervention is another big challenge for current mental health services. Sometimes, clinicians might miss the early signs of changes in a patients’ condition. Even with machine learning models that can identify the top features predictive of the symptoms trajectories, those top features are usually difficult for patients to act upon. Conversely, with rhythm features, machine learning will be able to provide patients with more actionable steps to help them stay in a stable mental condition. For example, as we know that the ultradian rhythm of patients’ movement behavior may impact their feeling of calm, the system can detect if there are significant changes in the ultradian rhythm of a patient’s movement behavior. If there is, the system can immediately prompt notification, reminding the user to try to calm down in a certain time period or devote themselves to different kinds of activities to ensure their movement rhythm to be stable.

### Limitations and future work

There are some limitations in this work. First, we used time-series features but our models are not temporal machine learning models. With extension to temporal models with time-series features, we might be able to improve model performance even more. In addition, the interactions of the features were not taken into account in our MTL-weight based clustering analysis since our models considered only the linear combinations of the features, but not the interactions of the features. Another limitation is that in this paper, we predicted only one day in the future; however, our models can be applied to predict symptoms a week or a month into the future.

Lastly, in this work, we evaluated the models using two different cross-validation procedures, cross-validation with models being evaluated on randomly held-out test data and cross-validation with models being evaluated on future unseen data. Both of the procedures have their own pros and cons. The former procedure can potentially mitigate the effect of temporarily adjacent data on evaluation results; however, it might overestimate the performance of models if certain events, abnormal behaviors for instance, only appear in the future data. On the contrary, the latter procedure simulates the real-life scenarios where models are only trained on data collected prior to model deployment. Therefore, the procedure might give less biased evaluation results if the distribution of the future data and the past data are very different; nonetheless, if certain patterns appear in the data for a period of time and those patterns happen to appear in both the past and the future data after a data split, it is likely to cause the procedure to overestimate models’ performance. For future work, other cross-validation procedures can be employed to further investigate how other different cross-validation procedures will potentially influence evaluation results.

## Methods

### Statement

All experiments and methods were performed in accordance with relevant guidelines and regulations. The study was approved by the Committee for Protection of Human Subjects at Dartmouth College and Institutional Review Board at North Shore-Long Island Jewish Health System. Participants provided informed consent. Patients were included in this study if they were ages 18 or older and had a chart diagnosis of schizophrenia spectrum disorder.

### Data collection

**Figure 10:**
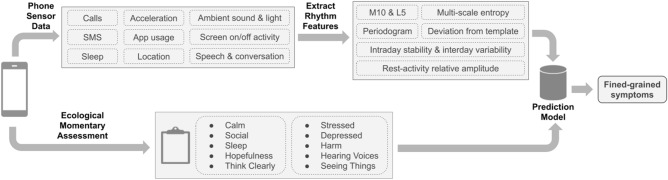
CrossCheck system overview.

Figure 10 illustrates the dataset we used for this study, which was collected by the CrossCheck system^11^. CrossCheck collected users’ passive sensing data continuously and prompted users to self-report their ecological momentary assessment (EMA) once every 2–3 days (Table 2). After filtering out participants who had less than 10 days of data, 61 participants with 6,152 days of EMA data remained (on average 104 days of data per participant) for modeling. Below we briefly introduce the dataset we used in this study. Please refer to our previous work^11^ for the details about the data collection.

#### Passive sensing data

- Acceleration: We collected 3-axis acceleration data from mobile phones, sampled from 50–100Hz. In the previous CrossCheck studies, we used the Android activity recognition API that includes: on foot, still, in vehicle, on bicycle, tilting, and unknown. However, in this paper, we extracted fine-grained activity rhythm features from raw acceleration.
- App usage: The Crosscheck system recorded the apps running on users’ phones every 15 min. We estimated *active app usage* by comparing app lists between every two consecutive sampling periods. The reason is that some apps may stay in the background even if the user is not actively using them. Specifically, we mapped each app to a category using meta-data from Google Play Store. We computed the number of apps being actively used during 24-h for each category. A total of 47 categories were recognized among all participants, with “NoneOrUtility” being the top category. This is not surprising as most of the system services were classified into this category. Among the other most common categories, we selected four of them for feature extraction: Communication, Entertainment, Productivity, and Social.
- Calls and SMS: We considered phone calls and SMS activities as indicators of the level of social interaction and communication. We logged incoming and outgoing calls and incoming and outgoing SMS.
- Screen on/off activity: User interaction with the phone is potentially indicative of general daily function and that can be captured through screen on/off activity. We logged timestamps of screen on and off events.
- Location: Prior studies have investigated the association between mobility patterns from geo locations and mental health^8,9,40^. In the context of schizophrenia, patients are found to be isolated and stay at home with little external contact, especially when experiencing distressing psychotic symptoms^12^. We logged trajectories of location from phones.
- Ambient environment: We logged ambient sound and light. The ambient sound reflects the ambient context of the participant’s acoustic environment, for example quiet isolated places versus noisy busy places. Similarly, the ambient light intensity also contains information about the environmental context of the participant, for example dark environment versus well-illuminated environment.
- Speech and conversation: Previous studies^6,8,41^ have shown that conversations and human voices are related to well-being and mental health. We detected human voices and conversational episodes using the previously developed algorithms^42^.
- Sleep: We computed the sleep-related features, which include sleep duration, bed time, and wake time during each day using a user’s screen and physical activity, ambient sound, and light^8,43^.

**Table 2:** Summary of the sensing data collected in the study.

Data type	Sensing data	Data description
Behavior	Acceleration	3-axis acceleration from mobile phone with sample rate of 50–100 Hz
	App usage	Number of apps used in the category of communication, entertainment, productivity, and social during every 15-min interval
	Call	Incoming and outgoing phone calls (and whether or not for incoming calls)
	SMS	Text message received (and whether they were read), sent, and drafted
	Screen on/off	Timestamps when screen was turned on and off
	Location	GPS (longitude and latitude) location of user
	Conversation	The onset and duration of conversation
	Sleep	Sleep duration, and bed and wake time
Environment	Light	The ambient light intensity collected using smartphone’s light sensor
	Sound	The volume of ambient sound

#### Self-reported EMA scores

EMA is a clinically validated method to capture states of mental health among people with schizophrenia^44^. The EMA used in this study consists of 10 one-sentence questions (Table 3), which are based on the self-reported dimensions defined in a previous schizophrenia study^45^. Patients were prompted to answer the EMA questions every 2–3 days by selecting a scale from 0 to 3 for each dimension.

**Table 3:** EMA questions used in the study.

Dimension	Description
Depressed	Have you been *DEPRESSED*?
Seeing things	Have you been *SEEING THINGS* other people can’t see?
Harm	Have you been worried about people trying to *HARM* you?
Hearing voices	Have you been bothered by *VOICES*?
Sleep	Have you been *SLEEPING* well?
Stressed	Have you been feeling *STRESSED*?
Think	Have you been able to *THINK* clearly?
Hopefulness	Have you been *HOPEFUL* about the future?
Social	Have you been *SOCIAL*?
Calm	Have you been feeling *CALM*?

Options: 0—not at all; 1—a little; 2—moderately; 3—extremely.

### Computing rhythm features

There are cyclic patterns, or rhythms, in human biological systems and behaviors to help humans respond to environmental influences^15^. Studies have shown the strong link between human rhythms and mental health^16,17,46^. The cyclic patterns of environmental influences, such as light and ambient noise, also have impacts on people’s mental health^35,47^. These rhythms have different periodicities, or recurring intervals. Based on whether the periodictiy is less than, equal to, or greater than 24 h, these rhythms can be characterized as ultradian, circadian, or infradian rhythm^35^, and the different periodicities influence people’s mental health in different ways^17,48,49^. As such, we applied a variety of metrics to patients’ sensing data in order to capture the change in their ultradian, circadian, and infradian rhythm respectively. These metrics include mean activity level during the most active 10 h and the least active 5 h, rest-activity relative amplitude, interday stability and intraday variability, deviation from template, multi-scale entropy, and periodogram. The type of rhythm each metric tries to capture is summarized in Table 4. We will describe the metrics in detail.

Another important thing that needs to be considered is the amount of historical data used for computing rhythms features. The pattern of these rhythms might change depending on the amount of historical data (window length) we are observing, for instance 2 days of data versus 14 days of data. Using different window lengths to compute the features will help us identify the most predictive window length to predict changes in symptoms in regard to different sensor modalities and different periodicities. For example, Reinertsen et al.^50^ showed that features extracted from heart-rate data and accelerometer data using an 8-day window generally resulted in higher accuracy for predicting schizophrenia than using a 2-day window. Taken together, we computed our rhythm features with the following procedure. First, for each type of sensor data, we took the data segment between day $d-w$ and day *d*, where *d* is the day when an EMA was reported and *w* is the window length. The values we used for window length are 2, 4, 6, 8, 10, 12, and 14 days. Next, for each data segment, we applied all the rhythm metrics (Table 4) to extract the rhythm features. As such, each rhythm feature entails information regarding (1) *modality*: the type of sensor data, (2) *periodicity*: which type of rhythm the metric corresponds to and (3) *time-window*: the amount of historical data used for extracting that feature, and. These three dimensions are summarized in Table 5. In the remainder of this section, we will describe all the rhythm metrics.

**Table 4:** The different rhythm categories and the corresponding rhythm metrics.

Periodicity	Rhythm Metrics
Ultradian	Multi-scale entropy, power spectrum density (with period less than 20 h)
Circadian	M10, L5, relative amplitude, deviation from template, interday stability, intraday variability, power spectrum density (with period greater than 20 h and less than 30 h)
Infradian	Power spectrum density (with period greater than 30 h)

The categorization, namely ultradian, circadian, and infradian, is based on whether the rhythm’s periodicity is less than, equal to, or greater than 24 h.

**Table 5:** The three dimensions used to characterize each feature and the different factors in each of the dimensions.

Dimension	Factor
Modality	Acceleration, app usage, call, SMS, screen on/off, location, conversation, sleep, light, sound
Periodicity	Ultradian, circadian, infradian rhythms
Time-window	Previous 2, 4, 6, 8, 10, 12 and 14 days

#### Multi-scale Entropy (MSE)

Multi-scale entropy^50^ is used to calculate a person’s sample entropy with a range of different time scales. Sample entropy (*SampEn*)^51^ is a measure of the complexity, or irregularity, of time series data, especially physiological time series^52,53^. Intuitively, it calculates the probability of finding matching templates with length $m+1$ (consecutive $m+1$ data points) given a matching template with length *m* and tolerance *r*. Mathematically, it is defined as:

1 $$\begin{aligned} SampEn(m, r, n) = -ln\frac{U^{m+1}}{U^m} \end{aligned}$$

where *m* is the template length, *r* is the tolerance, *n* is the total number of data points in the time series, and $U^m$ is the number of matching templates with length *m*.

Multiscale entropy calculates the *SampEn* for time series at less granular levels. More specifically, for the $\tau$-th time scale, each element of the coarse-grained time series, $y_j^\tau$, is given by:

2 $$\begin{aligned} y_j^\tau =\frac{1}{\tau }\sum _{i=(j-1)\tau +1}^{j\tau } x_i \end{aligned}$$

Sample entropy is then calculated for $y_j^\tau$ at time scale $\tau$.

It was suggested that $m=$ 1 or 2 and *r* in the range of 0.1–0.25 *S.D.* (standard deviation of the time series)^53^ tend to give good statistical properties. As such, we chose $m=2$ and $r=0.25\times S.D.$ when calculating the multiscale entropy. In this paper, we used 6 different time scales, $\tau$ = 1, 2, . . . , 6, which is aimed to capture the behavioral complexity in the time scale ranging from 1 to 6 h.

#### Power spectrum density

We applied periodograms to obtain the area under the curve of power spectrum density (PSD) with periods of 2, 4, 8, 16, 20, 22 h, 27, 28, 32, 36, 64, 72, 128, 256, and 512 h.

#### Most-active 10 h (M10) and least-active 5 h (L5)

The mean activity level during the most active 10 h, or *M10*, is defined as the mean value of the sensing data during the most active 10 consecutive hours. And the mean activity level during the 5 least active hours, or *L5*, is defined as the mean value of the sensing data during the least active 5 consecutive hours^54^. In general, M10 corresponds to a person’s daily activity and L5 corresponds to a person’s nocturnal activity. People with mental disorders or mental illnesses tend to have more irregular daily and nocturnal activity patterns, which results in more significant changes in their M10 and L5. In other words, the two values are proxies of a person’s level of activity during the day and at night, which can be used to detect changes in a person’s daily and nocturnal activity.

#### Rest-activity relative amplitude (RA)

Based on M10 and L5, rest-activity relative amplitude (RA) can be computed to characterize the difference between a person’s daily activity and nocturnal activity, which is calculated as:

3 $$\begin{aligned} RA = \frac{M10 - L5}{M10 + L5} \end{aligned}$$

The value of RA ranges from 0 to 1. Healthy people tend to have higher values of RA, which means increased activity during daytime and reduced activity during sleep, whereas people with mental disorder tend to have RA values that fluctuate quite a lot due to their irregular daily activity and sleep patterns.

#### Deviation from template

We computed 24-h activity templates (average hourly activities) with data from the past 2–14 days to capture short-term and long-term rhythm changes. Then we computed the mean, median, and standard deviation for the following two values: (1) the difference between the template and the hourly activity during each day for the past 2–14 days, and (2) the difference between the template and the previous day. These measures are indicators of rhythm changes over the past 2–14 days and any abrupt changes between the previous 2–14 days and the previous day.

#### Interday stability (IS) and intraday variability (IV)

Having a regular routine plays a huge role in the condition of people with mental disorder or mental illness. To quantify the regularity of a person’s routine over time, intraday stability and interday variability are the two important metrics that have been widely used^55^. Interday stability (IS), a measure of how stable a person’s rhythm is over multiple days, is defined as:

4 $$\begin{aligned} IS = \frac{n\sum _{h=1}^q({\overline{x}}_h-{\overline{x}})^2}{q\sum _{i=1}^n(x_i-{\overline{x}})^2} \end{aligned}$$

Intraday variability (IV) is a measure of the fragmentation of a person’s activity, namely how activity level shifts between two consecutive hours. It is defined as:

5 $$\begin{aligned} IV = \frac{n\sum _{i=2}^n(x_i-x_{i-1})^2}{(n-1)\sum _{i=1}^n(x_i-{\overline{x}})^2} \end{aligned}$$

where *n* is the total number of data points, $x_i$ represents individual data points, *q* is the total number of hours during the time frame of the measure, ${\overline{x}}_h$ is the hourly mean during hour *h*, and ${\overline{x}}=\frac{1}{n}\sum _{i=1}^nx_i$.

It is worth noting that IS and IV are only applicable to continuous time series. As such, we only applied IS and IV to participants’ accelerometer data, ambient light intensity, and ambient sound level.

### Feature benchmarking

Apart from our rhythm features, we also trained models with the features used in the previous studies^11,12^ to compare the resulting prediction accuracy. The features are summarized in Table 6.

**Table 6:** Summary of features used in previous work.

Features	Description
Physical activity	Durations of walking states, stationary state, and stationary plus in vehicle state
Speech and conversational interaction	The number of independent conversations and their duration as a proxy for social interaction. The ratio of detected human voice labels observed (e.g., amongst all inferred audio frames during a day, for example, 10% human voice)
Location and mobility	Distance traveled, the number of places visited, and location entropy from the location data, the number of places visited, the distance traveled, and location entropy using the centroid coordinates of visited places
Sleep	Sleep duration, sleep onset time, and wake time each 24 h period day based on the longest period of inferred sleep from ambient light, audio amplitude, activity, and screen on/off
Phone usage, calls, and texting	The number of phone lock/unlock events and the duration that the phone is unlocked. the number and duration of incoming and outgoing phone calls, and the number of incoming and outgoing SMS messaging
Ambient environment	Mean audio amplitude to determine the acoustic conditions ranging from quiet to loud environments. The standard deviation of the audio amplitude

### Algorithms

#### Single-task learning

Our first goal is to develop models that are more interpretable and clinically meaningful that clinicians can act on. To this end, beyond more interpretable rhythm features, we also need to reduce the feature dimensionality (originally 4157 features in total). We applied least absolute shrinkage and selection operator (LASSO), an effective way to select the most relevant features by adding regularization to control the sparsity of the coefficients in the model. Given the data $X_u\in {\mathbb {R}}^{e_u\times d}$ from user *u* (where $e_u$ is the number of EMA entries the user completed, and *d* is the dimension of the feature vector) and the user’s scores $Y_u^s\in {\mathbb {R}}^{e_u}$ for EMA symptom *s*, the objective function can be expressed as

6 $$\begin{aligned} \min _{w_u^s}\Vert Y_u^s-X_u w_u^s\Vert + \alpha \left| w_u^s \right| _1 \end{aligned}$$

$w_u^s\in {\mathbb {R}}^{d}$ is the weight vector for predicting symptom *s* for user *u*, and $\left| w_u^s\right| _1=\sum _{i}\left| w_{ui}^s\right|$ (the $l_1$-norm). $\alpha$ is the penalty for the sparsity of $w_u^s$. The bigger $\alpha$ we choose, the fewer non-zero coefficients $w_u^s$ will have and vice versa. Moreover, the absolute value of each element indicates how important that corresponding feature is in predicting scores of that EMA item.

If we assume that the feature weights for predicting a symptom *s* are identical across all the patients, we can also train a generalized model for predicting a score item *s* for all patients with similar formulation:

7 $$\begin{aligned} \min _{w^s}\Vert Y^s-X w^s\Vert + \alpha \left| w^s\right| _1 \end{aligned}$$

where $$X=\left[ X_1; \ldots ; X_p\right] $$$$\in {\mathbb {R}}^{(\sum _{u=1}^{p}e_u)\times d}$$, which is the concatenated feature matrix for user $$1,2,\cdots ,p$$; $$Y^s=[Y_1^s; \ldots ;Y_p^s]\in {\mathbb {R}}^{(\sum _{u=1}^{p}e_u)}$$, which is the concatenated EMA scores for symptom *s* across all the patients; and $$w^s \in {\mathbb {R}}^{d}.$$

#### Multi-task learning

From a clinical perspective, similar behavioral changes and trends that result in specific symptoms usually manifest in different users. Similarly, the different symptoms associated with schizophrenia do not happen independently^56^. When a patient experiences one symptom, hearing voices for instance, they might also experience other symptoms such as seeing things or feeling someone is going to harm them as a result of hallucination. However, the types or the extent of the symptoms might vary from patient to patient. Similarly, for different symptoms, some symptoms might be more severe than the others. As such, we need to have models that not only leverage information from different patients or related symptoms, but also account for individual or inter-symptom differences in order to best capture the key behavioral markers for the target patients or the target symptoms.

Multi-task learning^57^ (MTL) is a commonly used method to simultaneously train prediction models for multiple related tasks. MTL has been shown to train models that are better at capturing shared latent variables by taking into account not only the similarities but also the differences between the different tasks. In our case, the prediction tasks can be formulated as two different types of multi-task learning problems—Type (1): Given a user, predicting that user’s symptom scores for all the different symptoms; Type (2): Given a symptom, predicting all the users’ symptom scores for that symptom. For simplicity, we will use *MTL-symptoms* and *MTL-patients* to refer to these two MTL problems respectively.

Given a user’s EMA scores for all the different symptoms $Y_u=\left[ Y_u^1, \ldots , Y_u^k\right] \in {\mathbb {R}}^{e_u\times k}$, where *k* is the total number of different symptoms, the objective function for the MTL-symptoms, based on the formulations proposed by Nie et al.^24^ and Zhou et al.^26^, is formulated as

8 $$\begin{aligned} \min _{W_u}\left| \left| Y_u-X_uW_u\right| \right| _F^2 + \alpha \left| \left| W_u\right| \right| _{21} \end{aligned}$$

$W_u=\left[ W_u^1, \ldots , W_u^k\right] \in {\mathbb {R}}^{d\times k}$ is the weight matrix for predicting the scores for the individual symptoms. $\alpha$ is the regularization parameter. $\left| \left| \cdot \right| \right| _F$ is the Frobenius norm^58^. $\left| \left| W\right| \right| _{21} = \sum _i \sqrt{\sum _j w_{ij}^2}$ (the $l_{2,1}$-norm^59^). Joint $l_{2,1}$-norm minimization has been shown to be effective in enforcing joint group sparsity^21–23,25–27^. In other words, the regularization encourages a group of related tasks to share a small subset of features. We used Scikit-learn^60^, the open-source machine learning package, for the implementation.

For MTL-patients, the objective function for training all the users’ models to predict the scores for symptom *s*, which is based on the formulations proposed by Argyriou et al.^21,22^ and Zhou et al.^25^, is formulated as

9 $$\begin{aligned} \min _{W}\sum _{u=1}^p\left| \left| Y_u^s - X_uW_u^s\right| \right| _F^2+\alpha \left| \left| W^s\right| \right| _{21} \end{aligned}$$

where $W^s = \left[ W_1^s, \ldots , W_p^s\right] \in {\mathbb {R}}^{d\times s}$, and each column in $W^s$ is the weight vector for predicting each user’s scores for symptom *s*. The goal is to find a set of weight vectors that not only minimize the prediction error for each user, but also share as much commonality as possible.

For comparison, we also trained MTL models for MTL-symptoms using multi-output support least-squares vector regression machines^61^ (m-SVR). m-SVR is an extension of single-output support vector regression machine^62^ aimed to learn a mapping from multivariate input feature space to a multivariate output space. Instead of training multiple independent single-output SVRs for all the related tasks, this algorithm was proposed to learn $w_{0}$, the mean regressor for all the tasks, and $v_{i}$, a slight adjustment of the mean regressor when predicting the output for task *i*. In other words, the regressor for task $1, \ldots , T$ can be expressed as $w_{0}+v_{1}, \ldots , w_{0}+v_{T}$. The ultimate goal of this algorithm is to find small-sized vectors $v_{1}, \ldots , v_{T}$ to account for the task relatedness while minimizing the overall prediction error just like the optimization for single-output SVR.

### Experiment

#### Predicting EMA scores

We conducted an experiment to compare the performance of the following prediction models: (A) m-SVR with linear kernel, (B) m-SVR with radial basis function (RBF) kernel, (C) MTL-patients, (D) personalized MTL-symptoms, (E) generalized MTL-symptoms, (F) personalized STL, and (G) generalized STL. A personalized model means that a model is trained for each individual user using the individual’s data, whereas a generalized model means that a model is trained for all the users using all the users’ data.

To evaluate the performance of the models, we trained the individual models using the following manners to obtain the predicted EMA scores respectively.

*m-SVR models (for both types of m-SVR models)*: A m-SVR model was trained for each patient and predicted the scores for all the symptoms with fivefold cross validation. We got $e_u$ ($\#$ of EMA entries a patient completed)$\times$10 (symptoms) predicted scores for each patient after a fivefold cross validation, and in total $\sum _{u=1}^p(e_u\times 10)$ predicted scores across all the patients.

*MTL-patients models*: A model was trained for all the patients simultaneously for each symptom with semi leave-one-subject-out cross validation. That is, the test patient’s data was split into fivefolds; fourfolds of the data, along with other participants’ data, were used for training and the remaining data was used for testing. Hence, after repeating the process and using all folds of data for testing, we obtained $\sum _{u=1}^pe_u$ predicted scores (which is the total number of EMA entries completed by all the patients). In total, we got 10 (symptoms) $\times \sum _{u=1}^p e_u$ predicted scores.

*Personalized MTL-symptoms*: A model was trained for each patient to predict the scores for all the symptoms with fivefold cross validation. Therefore, we got $e_u$ ($\#$ of EMA entries a patient completed)$\times$10 (symptoms) predicted scores for each patient after a fivefold cross validation and in total $\sum _{u=1}^p(e_u\times 10)$ predicted scores for all the patients.

*Generalized MTL-symptoms:* One single model was trained for all the patients to predict the scores for all the symptoms with leave-one-subject-out cross validation. We obtained $\sum _{u=1}^p e_u\times 10$ predicted scores after a leave-one-subject-out cross validation.

*Personalized STL models:* A model was trained for each patient and each symptom with fivefold cross validation. Thus, we got $e_u$ predicted scores for each patient after a fivefold cross validation and in total $\sum _{u=1}^p(e_u\times 10)$ predicted scores.

*Generalized STL models:* A model was trained for all the patients to predict the scores for each symptom with leave-one-subject-out cross validation. Therefore, we got $\sum _{u=1}^p e_u$ predicted scores after a leave-one-subject-out cross validation and in total 10 (symptoms)$\times \sum _{u=1}^p e_u$ predicted scores.

With all the predicted scores for all the symptoms and for all the patients, we then computed and compared the root-mean-square-error (RMSE) of the predictions by each of the algorithms for each combination of symptom and patient. We conducted an aligned rank transform ANOVA^63^, with algorithm and symptom as the independent variables and RMSE as the dependent variable, to examine the main effects of algorithm and symptom. Post-hoc pairwise comparisons using Tukey’s Honest Significant Difference (HSD) test^64^ were also performed to compare the influences of the different algorithms.

In addition, we investigated how different cross-validation procedures might influence prediction accuracy, particularly procedures that simulate real clinical settings. As such, we trained and evaluated the models on historical and future data respectively. More specifically, we split the data into twofolds chronologically, with the first 80$\%$ of the data for training and the remaining 20$\%$ for testing. This can give us an idea about how well the models predict future EMA scores based on the historical data. For each model, we compared the resulting RMSEs to the RMSEs obtained from the cross-validation procedures mentioned earlier (e.g., randomly shuffled test sets for m-SVR(rbf) models and leave-one-subject-out cross-validation for MTL-patients models) using paired Wilcoxon tests^65^ and applied Holm-Bonferroni corrections^66^ to the individual comparisons to adjust the p-values.

Finally, we examined whether the rhythm features enabled better prediction performance compared to the traditional statistical features used in the previous studies^11,12^, where features computed over an entire day and over four different epoch periods—morning, afternoon, evening, and night, were used. We used the same experiment setup to train MTL-patients models with the same previously used statistical features (we chose MTL-patients models due to the models’ interpretability). We trained MTL-patients models to predict the individual symptoms with our proposed rhythm features and the traditional statistical features respectively; for each symptom, we compared the median difference in the RMSEs of models trained with rhythm and statistical features using paired Wilcoxon tests and applied Holm-Bonferroni corrections to adjust the p-values.

#### Finding heterogeneity

To better interpret how the different factors in each of the dimensions play a role in predicting different symptoms, we computed the mean aggregated weight for the individual factors in MTL-patient LASSO models. Essentially, each of the features is encoded with information from the three dimensions, sensor modality, periodicity, and window length, and there are several factors within each of the dimensions (Table 5). For a set of features $f_1, f_2, \ldots , f_n$ that entail information regarding factor *p* with corresponding weight $w_1, w_2, \ldots , w_n$, we computed the factor’s mean aggregated weight, or contribution, $c_p$ as $c_p=\frac{1}{n}\sum _{i=1}^n\left| w_i\right|$. It is worth noting that to make the results easier to interpret, for dimension *periodicity*, we first grouped the features based on ultradian, circadian, and infradian rhythm before we computed the contribution of the different factors.

#### Finding subtypes

In addition to heterogeneity in the top features for predicting different symptoms, we suspected that there are also individual differences in regard to how those different sensor modalities, rhythms, and window lengths play a role in predicting the same symptom. For example, some patients are more prone to change in the ultradian rhythm of the environmental noise^67^, and the same amount of change in the environmental noise is likely to have a significantly larger impact on their cognitive performance. That means that these patients’ models might have larger weights on specific sensor modalities, rhythms, or window lengths. To that end, we grouped the patients into different clusters based on the feature weights in their models in order to investigate if any subtypes exist.

For each symptom, we applied K-Means clustering algorithm^68^ to the feature weights across all the MTL-patients models to identify the different clusters, or the potential subtypes. Since we did not know the optimal number of subtypes for each symptom beforehand, we used *K* ranging from 2 to 10 for K-Means clustering and computed the corresponding mean silhouette score^69^ to determine the optimal number of clusters. Silhouette score is a metric to estimate the quality of clustering. The larger the mean silhouette score is, the better the data points are separated into different clusters. Once the optimal number of subtypes was determined, we then compared the top predictive features in the individual subtypes and the importance of different factors within each dimension.

## Supplementary information

Supplementary material 1

## Data Availability

The dataset generated during the current study is available from the corresponding author on reasonable request.

## References

[1] Patel KR, Cherian J, Gohil K, Atkinson D (2014). Schizophrenia: overview and treatment options. P & T Peer Rev. J. Form. Manag..

[2] Ben-Zeev D (2015). Mobile behavioral sensing for outpatients and inpatients with schizophrenia. Psychiatr. Serv..

[3] Firth, J. & Torous, J. Smartphone apps for schizophrenia: a systematic review. *JMIR mHealth uHealth* **3**, (2015). 10.2196/mhealth.4930 PMC4704940 26546039

[4] Torous, J. & Roux, S. Patient-driven innovation for mobile mental health technology: Case report of symptom tracking in schizophrenia. *JMIR Mental Health* **4**, (2017). 10.2196/mental.7911 PMC5519827 28684386

[5] Lane, N. D. *et al.* A survey of mobile phone sensing. *IEEE Commun. Mag.* **48**, (2010).

[6] Rabbi, M., Ali, S., Choudhury, T. & Berke, E. Passive and in-situ assessment of mental and physical well-being using mobile sensors. In *Proceedings of the 13th international conference on Ubiquitous computing*, 385–394 (ACM, 2011). 10.1145/2030112.2030164 PMC4180507 25285324

[7] Taylor, S. A., Jaques, N., Nosakhare, E., Sano, A. & Picard, R. Personalized multitask learning for predicting tomorrow’s mood, stress, and health. *IEEE Transactions on Affective Computing* (2017). 10.1109/TAFFC.2017.2784832 PMC7266106 32489521

[8] Wang, R. *et al.* Studentlife: assessing mental health, academic performance and behavioral trends of college students using smartphones. In *Proceedings of the 2014 ACM International Joint Conference on Pervasive and Ubiquitous Computing*, 3–14 (ACM, 2014).

[9] Saeb, S. *et al.* Mobile phone sensor correlates of depressive symptom severity in daily-life behavior: an exploratory study. *J. Med. Internet Res.* **17**, (2015). 10.2196/jmir.4273 PMC4526997 26180009

[10] Farhan, A. A. *et al.* Behavior vs. introspection: refining prediction of clinical depression via smartphone sensing data. In *Wirel. Health*, 30–37 (2016).

[11] Wang, R. *et al.* Crosscheck: toward passive sensing and detection of mental health changes in people with schizophrenia. In *Proceedings of the 2016 ACM International Joint Conference on Pervasive and Ubiquitous Computing*, 886–897 (ACM, 2016).

[12] Wang R (2017). Predicting symptom trajectories of schizophrenia using mobile sensing. Proc. ACM Interact. Mobile Wear. Ubiquitous Technol..

[13] Association, A. P. *et al.**Diagnostic and statistical manual of mental disorders (DSM-5®)* (American Psychiatric Pub, 2013). 10.1590/s2317-17822013000200017 24413388

[14] Kane JM (2012). Aripiprazole intramuscular depot as maintenance treatment in patients with schizophrenia: a 52-week, multicenter, randomized, double-blind, placebo-controlled study. J. Clin. Psychiatry.

[15] Aschoff, J. A survey on biological rhythms. In *Biological rhythms*, 3–10 (Springer, 1981).

[16] Wulff K, Dijk D-J, Middleton B, Foster RG, Joyce EM (2012). Sleep and circadian rhythm disruption in schizophrenia. Br. J. Psychiatry.

[17] Karatsoreos IN (2014). Links between circadian rhythms and psychiatric disease. Front. Behav. Neurosci..

[18] Ben-Zeev D (2014). Feasibility, acceptability, and preliminary efficacy of a smartphone intervention for schizophrenia. Schizophr. Bull..

[19] Ben-Zeev, D. *et al.* Mobile health (mhealth) versus clinic-based group intervention for people with serious mental illness: a randomized controlled trial. *Psychiatr. Serv.* appi–ps (2018). 10.1176/appi.ps.201800063 29793397

[20] Caruana R (1997). Multitask learning. Mach. Learn..

[21] Argyriou, A., Evgeniou, T. & Pontil, M. Multi-task feature learning. *Advances in neural information processing systems* **41–48** (2007).

[22] Argyriou A, Evgeniou T, Pontil M (2008). Convex multi-task feature learning. Mach. Learn..

[23] Liu, J., Ji, S. & Ye, J. Multi-task feature learning via efficient l 2, 1-norm minimization. In *Proceedings of the twenty-fifth conference on uncertainty in artificial intelligence*, 339–348 (AUAI Press, 2009).

[24] Nie, F., Huang, H., Cai, X. & Ding, C. H. Efficient and robust feature selection via joint 2, 1-norms minimization. *Adv. Neural Inf. Process. Syst.* **1813–1821**, (2010).

[25] Zhou, J., Chen, J. & Ye, J. Malsar: Multi-task learning via structural regularization. *Arizona State University* (2011).

[26] Zhou, J., Yuan, L., Liu, J. & Ye, J. A multi-task learning formulation for predicting disease progression. In *Proceedings of the 17th ACM SIGKDD international conference on Knowledge discovery and data mining*, 814–822 (2011).

[27] Zhou, J., Liu, J., Narayan, V. A. & Ye, J. Modeling disease progression via fused sparse group lasso. In *Proceedings of the 18th ACM SIGKDD international conference on Knowledge discovery and data mining*, 1095–1103 (2012). 10.1145/2339530.2339702 PMC4191837 25309808

[28] Ciliberto, C., Mroueh, Y., Poggio, T. & Rosasco, L. Convex learning of multiple tasks and their structure. *Int. Conf. Mach. Learn.* 1548–1557, (2015).

[29] Lu J (2018). Joint modeling of heterogeneous sensing data for depression assessment via multi-task learning. Proc. ACM Interact. Mobile Wear. Ubiquitous Technol..

[30] Van Dongen HP, Dinges DF (2005). Sleep, circadian rhythms, and psychomotor vigilance. Clin. Sports Med..

[31] Simon, E. B. & Walker, M. P. Sleep loss causes social withdrawal and loneliness. *Nat. Commun.* **9**, (2018). 10.1038/s41467-018-05377-0 PMC6092357 30108218

[32] Hall M, Donald P, Benedek M, Chang A (1996). Ultradian cycles of mood in normal and depressed subjects. Jefferson J. Psychiatry.

[33] Tracy JI (1996). Repetitive behaviors in schizophrenia: a single disturbance or discrete symptoms?. Schizophr. Res..

[34] Citrome L (2013). Addressing the need for rapid treatment of agitation in schizophrenia and bipolar disorder: focus on inhaled loxapine as an alternative to injectable agents. Ther. Clin. Risk Manag..

[35] Van Kamp, I. & Davies, H. Environmental noise and mental health: Five year review and future directions. In *Proceedings of the 9th international congress on noise as a public health problem* (2008).

[36] Wright B, Peters E, Ettinger U, Kuipers E, Kumari V (2016). Effects of environmental noise on cognitive (dys) functions in schizophrenia: A pilot within-subjects experimental study. Schizophr. Res..

[37] Lin I-M, Peper E (2009). Psychophysiological patterns during cell phone text messaging: A preliminary study. Appl. Psychophysiol. Biofeedback.

[38] Hudson, H. K., Bliss, K. R. & Fetro, J. V. Effects of text messaging on college students’ perceptions of personal health. *Health Educ.* **44**, 28–35 (2012).

[39] Murdock, K. K. Texting while stressed: Implications for students’ burnout, sleep, and well-being. *Psychol. Popular Media Cult.* **2**, 207 (2013).

[40] Canzian, L. & Musolesi, M. Trajectories of depression: unobtrusive monitoring of depressive states by means of smartphone mobility traces analysis. In *Proceedings of the 2015 ACM international joint conference on pervasive and ubiquitous computing*, 1293–1304 (ACM, 2015).

[41] Lane, N. D. *et al.* Bewell: A smartphone application to monitor, model and promote wellbeing. In *5th international ICST conference on pervasive computing technologies for healthcare*, 23–26 (2011).

[42] Wyatt, D. M. *et al.**Measuring and modeling networks of human social behavior* (Citeseer, 2010).

[43] Chen, Z. *et al.* Unobtrusive sleep monitoring using smartphones. In *Pervasive Computing Technologies for Healthcare (PervasiveHealth), 2013 7th International Conference on*, 145–152 (IEEE, 2013).

[44] Granholm E, Loh C, Swendsen J (2007). Feasibility and validity of computerized ecological momentary assessment in schizophrenia. Schizophr. Bull..

[45] Ben-Zeev D, McHugo GJ, Xie H, Dobbins K, Young MA (2012). Comparing retrospective reports to real-time/real-place mobile assessments in individuals with schizophrenia and a nonclinical comparison group. Schizophr. Bull..

[46] Li JZ (2013). Circadian patterns of gene expression in the human brain and disruption in major depressive disorder. Proc. Nat. Acad. Sci..

[47] Bedrosian T, Nelson R (2017). Timing of light exposure affects mood and brain circuits. Transl. Psychiatr..

[48] De Graaf R, Van Dorsselaer S, Ten Have M, Schoemaker C, Vollebergh WA (2005). Seasonal variations in mental disorders in the general population of a country with a maritime climate: findings from the netherlands mental health survey and incidence study. Am. J. Epidemiol..

[49] Blum, I. D. *et al.* A highly tunable dopaminergic oscillator generates ultradian rhythms of behavioral arousal. *Elife* **3** (2014). 10.7554/eLife.05105 PMC4337656 25546305

[50] Reinertsen E (2017). Continuous assessment of schizophrenia using heart rate and accelerometer data. Physiol. Meas..

[51] Richman, J. S., Lake, D. E. & Moorman, J. R. Sample entropy. In *Methods in enzymology*, vol. 384, 172–184 (Elsevier, 2004). 10.1016/S0076-6879(04)84011-4 15081687

[52] Richman JS, Moorman JR (2000). Physiological time-series analysis using approximate entropy and sample entropy. Am. J. Physiol. Heart Circ. Physiol..

[53] Jia Y, Gu H, Luo Q (2017). Sample entropy reveals an age-related reduction in the complexity of dynamic brain. Sci. Rep..

[54] Gonçalves BS, Cavalcanti PR, Tavares GR, Campos TF, Araujo JF (2014). Nonparametric methods in actigraphy: An update. Sleep Sci..

[55] Witting, W., Kwa, I., Eikelenboom, P., Mirmiran, M. & Swaab, D. Alterations in the circadian rest-activity rhythm in aging and alzheimer’s disease. *Biol. Psychiatr.* **27**, 563–572 (1990). 10.1016/0006-3223(90)90523-5 2322616

[56] Kumar V, Bagewadi V, Sagar D, Varambally S (2017). Multimodal hallucinations in schizophrenia and its management. Indian J. Psychol. Med..

[57] Evgeniou, T. & Pontil, M. Regularized multi–task learning. In *Proceedings of the tenth ACM SIGKDD international conference on Knowledge discovery and data mining*, 109–117 (ACM, 2004).

[58] Van Loan CF, Golub GH (1983). Matrix computations.

[59] Yang Y, Shen HT, Ma Z, Huang Z, Zhou X (2011). l2, 1-norm regularized discriminative feature selection for unsupervised learning. IJCAI Proc. Int. Joint Conf. Artif. Intell..

[60] Pedregosa F (2011). Scikit-learn: machine learning in python. J. Mach. Learn. Res..

[61] Xu S, An X, Qiao X, Zhu L, Li L (2013). Multi-output least-squares support vector regression machines. Pattern Recogn. Lett..

[62] Drucker, H., Burges, C. J., Kaufman, L., Smola, A. J. & Vapnik, V. Support vector regression machines. *Adv. Neural Inf. Process. Syst.* 155–161, (1997).

[63] Wobbrock, J. O., Findlater, L., Gergle, D. & Higgins, J. J. The aligned rank transform for nonparametric factorial analyses using only anova procedures. In *Proceedings of the SIGCHI conference on human factors in computing systems* 143–146, (2011).

[64] Jaccard J, Becker MA, Wood G (1984). Pairwise multiple comparison procedures: a review. Psychol. Bull..

[65] Gehan EA (1965). A generalized wilcoxon test for comparing arbitrarily singly-censored samples. Biometrika.

[66] Abdi, H. Holm’s sequential bonferroni procedure. *Encyclopedia of research design* **1**, 1–8 (2010).

[67] Stansfeld SA (1992). Noise, noise sensitivity and psychiatric disorder: epidemiological and psychophysiological studies. Psychol. Med. Monogr. Suppl..

[68] Basu, S., Banerjee, A. & Mooney, R. Semi-supervised clustering by seeding. In *In Proceedings of 19th International Conference on Machine Learning (ICML-2002* (Citeseer, 2002).

[69] Rousseeuw PJ (1987). Silhouettes: a graphical aid to the interpretation and validation of cluster analysis. J. Comput. Appl. Math..

