# Supplementary material for: Using behavioral rhythms and multi-task learning to predict fine-grained symptoms of schizophrenia
Source: Sci Rep. 2020 Sep 15;10:15100. doi: 10.1038/s41598-020-71689-1 (PMC7492221; doi:10.1038/s41598-020-71689-1)
Supplement: Supplementary file 1 — Supplementary material 1 [file 41598_2020_71689_MOESM1_ESM.pdf]

Using Behavioral Rhythms and Multi-task Learning to Predict Fine Grained Symptoms of Schizophrenia

Vincent W.-S. Tseng[1,*,+], Akane Sano[2,+], Dror Ben-Zeev[3], Rachel Brian[3], Andrew T. Campbell[4], Marta Hauser[5], John M. Kane[6], Emily A. Scherer[7], Rui Wang[8], Weichen Wang[4], Hongyi Wen[1], and Tanzeem Choudhury[1]

[1]Cornell University, Information Science, Ithaca, 14850, USA
[2]Rice University, Department of Electrical and Computer Engineering, Houston, 77005, USA
[3]University of Washington, Psychiatry and Behavioral Sciences, Seattle, 98195, USA
[4]Dartmouth College, Computer Science, Hanover, 03755, USA
[5]Vanguard Research Group, USA
[6]The Donald and Barbara School of Medicine at Hofstra/ Northwell, Department of Psychiatry, Hempstead, 11549, USA
[7]Dartmouth Geisel School of Medicine, Biomedical Data Science Department, Hanover, 03755, USA
[8]Facebook, Inc., USA
[*]wt262@cornell.edu
[+]these authors contributed equally to this work

Supplementary Information

Ranking	**Depressed**	**Seeing Things**
1	#draft_messages⊗8-hour_PSD⊗2-day_window	light⊗medain_deviation⊗14-day_window
2	#draft_messages⊗10-hour_PSD⊗2-day_window	ambient_sound⊗3-hour_MSE⊗12-day_window
3	ambient_sound⊗3-hour_MSE⊗6-day_window	ambient_sound⊗5-hour_MSE⊗10-day_window
4	#draft_messages⊗2-hour_PSD⊗2-day_window	ambient_sound⊗5-hour_MSE⊗8-day_window
5	#draft_messages⊗27-hour_PSD⊗2-day_window	ambient_sound⊗1-hour_MSE⊗2-day_window
6	#draft_messages⊗12-hour_PSD⊗2-day_window	ambient_sound⊗5-hour_MSE⊗6-day_window
7	ambient_sound⊗3-hour_MSE⊗12-day_window	#outgoing_calls⊗4-hour_PSD⊗2-day_window
8	ambient_sound⊗5-hour_MSE⊗10-day_window	#missed_calls⊗4-hour_PSD⊗8-day_window
9	#incoming_calls⊗4-hour_PSD⊗6-day_window	ambient_sound⊗2-hour_MSE⊗10-day_window
10	ambient_sound⊗5-hour_MSE⊗8-day_window	#outgoing_calls⊗SD_deviation⊗14-day_window

Ranking	**Harm**	**Hearing Voices**
1	ambient_sound⊗3-hour_MSE⊗12-day_window	ambient_sound⊗3-hour_MSE⊗6-day_window
2	ambient_sound⊗5-hour_MSE⊗10-day_window	#draft_messages⊗10-hour_PSD⊗2-day_window
3	#incoming_calls⊗4-hour_PSD⊗6-day_window	#draft_messages⊗12-hour_PSD⊗2-day_window
4	ambient_sound⊗5-hour_MSE⊗8-day_window	light⊗median_deviation⊗14-day_window
5	ambient_sound⊗5-hour_MSE⊗6-day_window	ambient_sound⊗3-hour_MSE⊗12-day_window
6	ambient_sound⊗1-hour_MSE⊗12-day_window	#draft_messages⊗2-hour_PSD⊗2-day_window
7	#outgoing_calls⊗4-hour_PSD⊗2-day_window	#draft_messages⊗8-hour_PSD⊗2-day_window
8	ambient_sound⊗4-hour_MSE⊗8-day_window	ambient_sound⊗5-hour_MSE⊗10-day_window
9	#missed_calls⊗4-hour_PSD⊗8-day_window	#incoming_calls⊗4-PSD_MSE⊗6-day_window
10	ambient_sound⊗2-hour_MSE⊗10-day_window	ambient_sound⊗5-hour_MSE⊗8-day_window

Ranking	**Sleep**	**Stressed**
1	light⊗median_deviation⊗14-day_window	#draft_messages⊗8-hour_PSD⊗2-day_window
2	#outgoing_calls⊗SD_deviation⊗14-day_window	#draft_messages⊗10-hour_PSD⊗2-day_window
3	ambient_sound⊗3-hour_MSE⊗6-day_window	#draft_messages⊗2-hour_PSD⊗2-day_window
4	#draft_messages⊗8-hour_PSD⊗2-day_window	#draft_messages⊗12-hour_PSD⊗2-day_window
5	#draft_messages⊗64-hour_PSD⊗2-day_window	ambient_sound⊗3-hour_MSE⊗12-day_window
6	ambient_sound⊗3-hour_MSE⊗12-day_window	#incoming_calls⊗4-hour_PSD⊗6-day_window
7	#draft_messages⊗10-hour_PSD⊗2-day_window	ambient_sound⊗1-hour_MSE⊗2-day_window
8	#draft_messages⊗27-hour_PSD⊗2-day_window	ambient_sound⊗5-hour_PSD⊗10-day_window
9	screen_on_time⊗SD_deviation⊗10-day_window	ambient_sound⊗5-hour_PSD⊗8-day_window
10	#draft_messages⊗12-hour_PSD⊗2-day_window	#draft_messages⊗6-hour_PSD⊗2-day_window

Ranking	**Think**	**Hopeful**
1	light⊗median_deviation⊗14-day_window	#draft_messages⊗8-hour_PSD⊗2-day_window
2	#draft_messages⊗8-hour_PSD⊗2-day_window	light⊗median_deviation⊗14-day_window
3	#draft_messages⊗10-hour_PSD⊗2-day_window	#draft_messages⊗10-hour_PSD⊗2-day_window
4	#draft_messages⊗2-hour_PSD⊗2-day_window	#draft_messages⊗12-hour_PSD⊗2-day_window
5	#draft_messages⊗12-hour_PSD⊗2-day_window	#draft_messages⊗2-hour_PSD⊗2-day_window
6	ambient_sound⊗3-hour_MSE⊗6-day_window	ambient_sound⊗3-hour_MSE⊗12-day_window
7	#outgoing_calls⊗SD_deviation⊗14-day_window	#draft_messages⊗27-hour_PSD⊗2-day_window
8	ambient_sound⊗3-hour_MSE⊗12-day_window	#draft_messages⊗64-hour_PSD⊗2-day_window
9	#draft_messages⊗64-hour_PSD⊗2-day_window	ambient_sound⊗3-hour_MSE⊗6-day_window
10	#draft_messages⊗6-hour_PSD⊗2-day_window	ambient_sound⊗4-hour_MSE⊗8-day_window

Ranking	**Social**	**Calm**
1	light⊗median_deviation⊗14-day_window	light⊗median_deviation⊗14-day_window
2	#outgoing_calls⊗SD_deviation⊗14-day_window	ambient_sound⊗3-hour_MSE⊗6-day_window
3	ambient_sound⊗3-hour_MSE⊗6-day_window	ambient_sound⊗3-hour_MSE⊗12-day_window
4	ambient_sound⊗3-hour_MSE⊗12-day_window	#outgoing_calls⊗SD_deviation⊗14-day_window
5	#outgoing_calls⊗SD_deviation⊗14-day_windo	#draft_messages⊗10-hour_PSD⊗2-day_window
6	#missed_calls⊗12-hour_PSD⊗8-day_window	light⊗3-hour_MSE⊗2-day_window
7	ambient_sound⊗5-hour_MSE⊗2-day_window	#draft_messages⊗8-hour_PSD⊗2-day_window
8	light⊗3-hour_MSE⊗2-day_window	#outgoing_calls⊗SD_deviation⊗14-day_window
9	conversation_length⊗SD_deviation⊗10-day_window	#outgoing_calls⊗mean_deviation⊗14-day_window
10	#incoming_calls⊗4-hour_PSD⊗14-day_window	conversation_length⊗SD_deviation⊗10-day_window

Table S1. Top predictive features for different schizophrenia symptoms.
